# Aerospace Environmental Challenges for Electrical Insulation and Recent Developments for Electrified Aircraft

**DOI:** 10.3390/ma15228121

**Published:** 2022-11-16

**Authors:** Maricela Lizcano, Tiffany S. Williams, Euy-Sik E. Shin, Diana Santiago, Baochau Nguyen

**Affiliations:** 1 NASA Glenn Research Center, Cleveland, OH 44135, USA; 2 Universities Space Research Association, Cleveland, OH 44135, USA

**Keywords:** aerospace electrical insulation, extreme environments, high voltage, Micro-Multilayered Multifunctional Electrical Insulation (MMEI), electrical insulation composites, hexagonal boron nitride (h-BN)

## Abstract

The growing trend towards high voltage electrical assets and propulsion in the aeronautics and space industry pose new challenges in electrical insulation materials that cannot be overlooked. Transition to new high voltage electrified systems with unprecedented high levels of voltage, power, and efficiency must be safe and reliable. Improvements in both performance and safety of megawatt power systems is complicated because of the need for additional power transmission wiring and cabling and new safety requirements that have the potential of making the resulting systems heavier. To mitigate this issue, novel lightweight materials and system solutions are required that would result in lower specific weights in the insulator and conductor. Although reduced size and weight of system components can be achieved with new concepts, designs, and technologies, the high voltage (≥300 V) operation presents a significant challenge. This challenge is further complicated when considering the extreme operating environment that is experienced in aircraft, spacecraft, and targeted human exploration destinations. This paper reviews the extreme environmental challenges for aerospace electrical insulation and the needs associated with operating under high voltage and extreme environments. It also examines several recently developed robust lightweight electrical insulation materials that could enhance insulation performance and life. In aerospace, research must consider mass when developing new technologies. The impact of these recent developments provides a pathway which could enable next generation high altitude all electric aircraft, lightweight power transmission cables for a future sustained presence on the Moon and missions to Mars using HV propulsion, such as spacecraft with Nuclear Electric Propulsion systems.

## 1. Introduction

The increasing need for power and clean energy is pushing technological development in the aeronautics and space sector toward electrified propulsion systems, such as hybrid-electric propulsion in aircraft, nuclear electric power for spacecraft, and power transmission cables for sustained lunar exploration. This trend today, and the rapid pace of technological development, is not necessarily new, at least not for terrestrial transportation. Electric propulsion and power transmission in modern terrestrial vehicles finds its roots as far back as the late 1800s [1]. Although Krebs and Renard used an electric motor and batteries to fly an airship [2], it was not until 1973 that electrified aircraft propulsion (EAP) was first debuted by Fred Militky [3]. He demonstrated the feasibility of a manned EAP, by converting a motor glider to electric power using Ni–Cd batteries. The leap to bring high-power systems to aeronautics and space applications seems to be an obvious progression for clean energy solutions and has promoted the feasibility of a new era in aerospace systems utilizing high voltage (HV). For this discussion, high voltage refers to voltages of >300 V, as the current space application at ≤300 V has been explored extensively.

Aeronautic trends towards HV are driven by sustainable air mobility growth, clean energy, and climate change solutions. Increasing fuel efficiency, reducing noise and reducing pollution from harmful carbon dioxide (CO_2_) and nitrogen oxides (NOx) emissions helps to address climate concerns and sustainable air mobility [4]. More electric aircraft (MEA), such as Boeing’s 787, have reduction in weight due to replacing pneumatic and mechanical systems with electrical systems, saving on fuel consumption [5,6]. As for space applications, the trends are driven by larger power demands, mass and volume constraints, and cost of heavy launch payload [7].

One technological area that has more recently gained attention is in new aerospace HV power cables and wires. Increasing power requires increasing voltage and/or current in transmission systems. The wiring system large transport aircraft can easily be hundreds of miles long with a total weight of over 12,000 lbs [5]. Compared to other aerospace electrical components, such as electric machines, power electronics, and inverters, the cables and wiring have lagged in materials development [8]. Space applications can also be expected to increase in power needs, thus, increasing the requirement for more electrical wiring resulting in a corresponding increase in total specific weight. Increasing power demands can be achieved by using HV assets, allowing for reduction in conductor size, and, therefore, lowering the conductor weight. However, HV usually requires thicker electrical insulation and protection from electric fields, since HV increases the possibility of arcing events and flashovers [9], which can diminish the benefit of a lower conductor weight. Furthermore, at HV, current aerospace insulation materials can experience more operational thermal, electrical and mechanical stresses [10]. The challenges are not easily overcome. The paradigm of mass and volume constraints and HV operation requires careful component design, advanced materials, and thoughtful materials processing methods [7,11,12]. Novel lightweight electrical insulation architectures and nano materials could provide robust solutions.

This paper focuses on the challenges associated with operating HV aerospace assets in extreme environments and presents recent research developments in electrical insulation for power transmission systems in electrified aircraft. The environmental challenges are related to effects of high altitude, Lunar surface exosphere, and manned space missions.

## 2. Electrical Discharges and Extreme Environments

**Figure 1:**
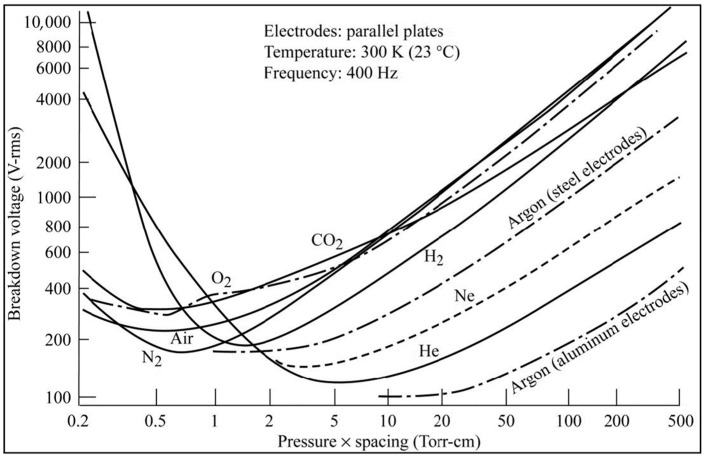
Dielectric breakdown Paschen curves for various gases at room temeprature and 400 Hz.

Part of the design criteria for aerospace electrical and electronic technologies is based on Paschen’s Law [7,11,13]. Paschen’s Law describes the voltage breakdown of gases between two conductors in an electric field as a function of the product of pressure and the gap distance between the two conductors. The main challenges associated with today’s high-power electrified aircraft needs are that the voltage for safe design in air is typically less than 327 V, the Paschen threshold for air [11,13,14]. Figure 1 [13] below shows the breakdown voltage of different gases at room temperature and AC frequency of 400 Hz as a function of the product of environmental pressure and the gap distance between two adjacent conductors, showing the presence of a critical minimum for all gases to be less than 400 V.

Broadly speaking, when a gas is exposed to a high energy electric field strong enough to polarize gas molecules, the molecules can be accelerated [9,11,13]. Colliding gas molecules may become ionized. The ionized gas and electrons begin to build up on surfaces, developing a potential, and, eventually, resulting in an arcing event, causing damage. Corona discharges can also arise in high electric fields in a localized gaseous medium [11,13]. When electrical discharge between two conductors is bridged, electrical breakdown occurs [13].

Partial discharge (PD) is another phenomenon of consequence which usually occurs when the electric discharge between two conductors is not bridged. It occurs inside insulating materials as the result of voids, cracks, contamination, aging, defects (either inherent in the material or from manufacturing processes), and maintenance [7,11,12,14,15]. Gases inside of voids experience PD based on many factors, such as applied voltage, voltage rise rate, electric field strength, thermal and mechanical environmental stresses, space charge, interfaces, insulation thickness, void sizes and shapes, and insulation type [12,13,15,16]. PD is particularly critical. Once PD inception voltage (PDIV) is reached inside existing defects, the bonds of the insulating medium begin to break and continue to grow with more PD, further degrading the material. PD effectively becomes an extrinsic aging factor. The insulation aging, resulting in PD, is also accelerated by increased PD rate until insulation failure eventually occurs [17,18].

A critical factor affecting insulation is space charge [16], contributing to degradation and breakdown of insulating materials. Space charge can alter, amplify, or lower the local electric field inside a void. Space charge can also increase aging rate that can result in high energy degradation processes causing premature insulation failure. The influence of space charge is related to many parameters, such as insulating and conductor material, the electrical stresses strength, temperature, material impurities or additives and interfaces. Furthermore, it is not yet known how space charge contributes to insulation degradation. [16,18,19]. It is, therefore, prudent to include comprehensive study of space charge. New insulation development should include processing methods that can, to a great extent, eliminate contamination, defects, and voids during laboratory and manufacturing processes [12]. This can eliminate some problems with electrical discharge phenomenon and space charge; however, this alone is not sufficient. A synergistic community effort conducted thorough studies promotes insight for new HV electrical insulation candidates.

### 2.1. High Voltage High Altitude Flight

**Figure 2:**
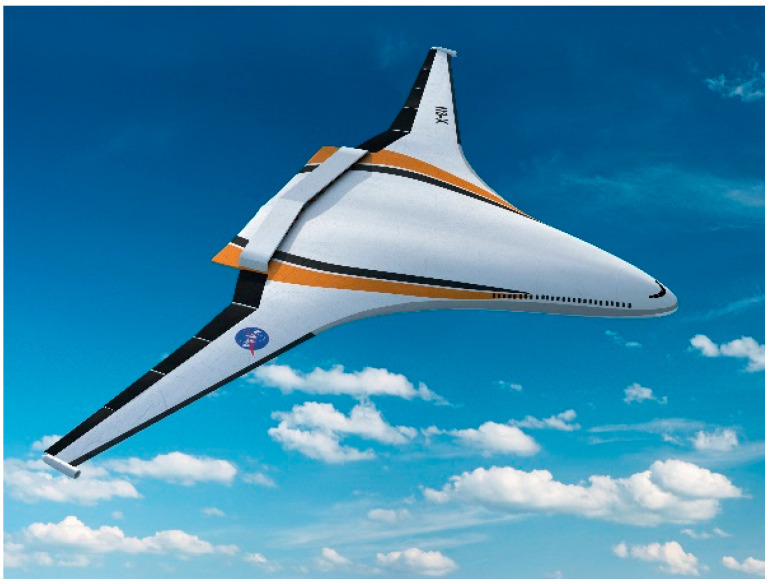
NASA’s N3-X, Turboelectric blended wing body concept.

Altitude must be considered for HV operations, as it poses significant safety concerns for applications with voltages exceeding 300 V, according to Paschen’s Law [13]. The main challenges are a combination of operating at high altitude (low pressures) under high voltages, currents, and frequencies, where arcing events are more prone to occur, while keeping insulation mass and volume low [9]. The compact nature of electrical wiring inside an aircraft makes it difficult to increase the gap between two adjacent conductors, and low pressure at a certain gap distance can create an unsafe discharging event [12]. As an example of a future large subsonic aircraft, NASA’s Turboelectric concepts, shown in Figure 2 [20], is an aircraft with high power needs that would require HV operations. These systems would likely run a combination of AC and DC voltages. This aircraft has been described as a megawatt class turboelectric system and was highlighted during NASA’s Convergent Aeronautics Solutions (CAS) High Voltage Hybrid Electric Propulsion (HVHEP) project. In the CAS study, the electrical system was based on an HV, 3-phase AC power system comprised of a double fed induction machine [21]. The high-power transmission electrical insulation needs to perform under variable frequencies, *f* = 400 to 4000 Hz, with a maximum voltage at lift-off of 20 kV and 10 kV at cruising speeds. In this case, power levels are for ≥10–20 MW [21,22,23]. At HV, there is an increased chance of corona discharge, as well as increased chances of PD in insulation [24].

The main environmental challenge for HV is the low pressure. As a large aircraft ascends to cruising speeds and high altitudes, the density of air is greatly reduced, due to the low pressure. In these conditions, the low-density air molecules are more easily accelerated in high voltage electric fields. Further, air molecule collisions continue to occur, as well as secondary collisions. At certain pressure and conductor gap distance, charge build up on conductor surfaces can surpass a threshold potential and eventually cause breakdown [13]. In other words, the low-pressure conditions make corona phenomenon more likely to occur, while the HV makes insulation more vulnerable to PD, decreasing insulation life, which can lead to catastrophic events [11,25].

In steady state DC field, PD repetition rates are lower than in cables operating under AC fields. Nevertheless, compared to an AC voltage supply, steady-state DC cables experience voltage transients which can last a significant amount of time before steady state is achieved [17,18]. During voltage transients, the electric field distribution in the insulation and voids depends on the insulation’s electrical conductivity and permittivity. DC voltage transients can cause PD inside of material voids which do not usually occur during DC steady state. Further, at the beginning of a voltage transient, PD repetition rates are similar to those of AC. Depending on temperature, repetition rates can take hours to decrease to low repetition rates. Over time, this could cause premature insulation failure. At some point, PD inception voltage decreases [17,18]. Voltage transients and faults are not limited to transmission cables, since other electrical systems, such as power electronics and converters, can experience catastrophic arcing events as well. These conditions become critical when operating at high altitudes and high voltage, emphasizing the need for new insulation solutions.

### 2.2. The Lunar Environment

NASA’s goal for a sustained human presence on the Moon requires the development of novel power transmission materials as well. Advances in mission-enabling cables/wires currently trail that of other power distribution technologies.

**Figure 3:**
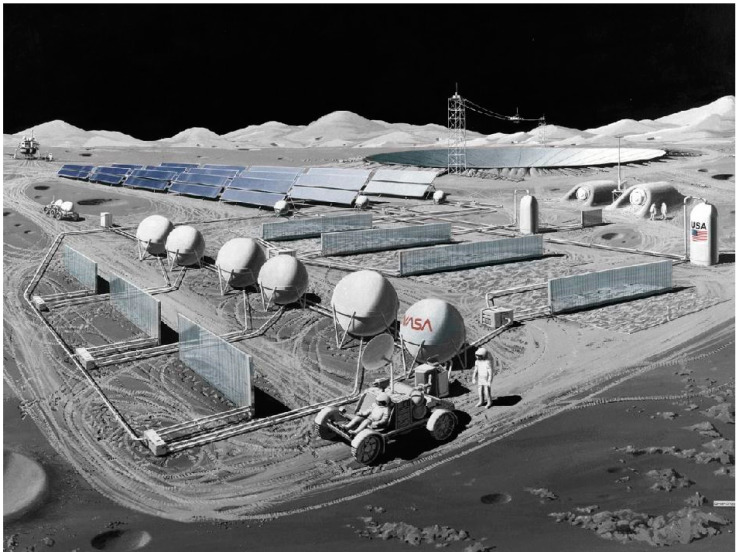
Concept of manned lunar observatory.

Three-phase AC power and/or DC transmission are key enabling technologies for high voltage (HV) Lunar power transmission systems capable of transmitting 100 kW between distal assets within the extreme Lunar environment [26,27]. As an increasing number of public and private entities set their sights towards a future on the Moon, the demand for surface power might dramatically increase, potentially up to megawatt levels [27,28]. This power is critical for missions supporting sustained human presence, new science exploration activities, and the construction of large-scale infrastructure. Figure 3, for example, shows a lunar observatory concept [29].

Terrestrial, state-of-the-art (SOA) cables for high-voltage, high-power applications easily exceed a weight/unit ratio of 1.0 kg/m [30,31]. For the Moon application, transmission cables that are almost half that weight. at approximately 0.55 kg/m, are desired [32]. Connectors, joining systems, and spools further increase the subsystem mass. Thus, there is a critical need to develop lightweight, low-mass and volume materials for power transmission cables/wires that are safe, effective, and reliable in the lunar environment. Trade studies recommend AC power transmission in a range of 400 Hz to 1 kHz at 3 kV for efficiency and to reduce mass [28]. If AC cables are used, the development of multifunctional lightweight conductor electrical insulation would greatly benefit power transmission performance and reliability and meet NASA’s desired cable weight goals.

Unlike Earth, the lunar environment presents significant challenges that would contribute to space weathering [33] of power transmission cables/wires and could cause premature aging. Other challenges include: (1) galactic cosmic and UV radiation exposure, as well as solar energetic particles (SEPs) [33,34,35]; (2) extreme temperatures swings [36,37]; (3) the energized lunar exosphere; and (4) the electrostatic and abrasive lunar regolith [36,38]. It may be possible to bury cables under the lunar regolith. However, this raises other issues with deployment, maintenance, and condition monitoring. Understanding the degradation due to the lunar environment and the effects on power transmission materials aids in advancing insulation technology towards high performance reliability.

A closer look at the lunar environment indicates that cosmic radiation, UV rays, solar wind, and solar energetic particles (SEPs) can all be harmful to assets on the Moon. During the lunar day cycle the regolith, through photoelectric emissions, charges and ionizes regolith particles. The particles on the lunar day side are positively charged, due to the photoelectron emissions, while particles on the night side are negatively charged [39,40,41]. Even more interesting, is the voltage potential (V) on the lunar surface that can depend on different factors, such as location on the Moon. For example, on the day side, the lunar surface has potential less than 20 V, while the dark side has potential ranging from −100 V to −4 kV [42].

The regolith can experience dielectric breakdown due to the UV component of sunlight [33,41]. Here are some considerations. The lunar regolith particles are made of different materials and dielectric medium. In one example, [43] describes that SEP exposure on permanently shaded areas causes dielectric breakdown of the lunar regolith. Arcing events can occur along the particle’s grain boundaries where jagged edges can locally intensify the electric fields by 1–2 orders of magnitude, followed by breakdown of the particles caused by micro-electric events. The electric field can also intensify at the grain boundaries, due to the difference in dielectric properties between particles’ interface boundaries, causing breakdown. Moreover, the particles can have inclusions, voids, and pockets of gas (>3 microns). The gases are a result of either meteor impacts or impregnation by solar wind [33,44,45]. Although SEP is mostly related to regolith breakdown on the shaded Moon, the point is that the regolith can breakdown with sufficient energy. For example, these pockets of gases can be thought of as voids in insulation. The ionization of the interior gases causes micro explosions and electrical breakdown. This means that if a conductor is laid on the regolith, with or without electrical insulation, the energized conductor could intensify local fields in the regolith and induce regolith breakdown at the regolith–cable interfaces. Over time, the conductor would experience power losses due to micro defects along its length and abrasions on its surface, which is likely to reduce the conductor efficiency or cable life. New electrical insulation solutions should withstand the breakdown impact and exploding fragmented regolith.

The extreme temperature cycles on the Moon must be considered in designing electrical insulation. The Moon’s daylight cycles are much longer than those of the Earth. The Sun shines on the Moon for 14.5 Earth days and is hidden from the sun for another 14.5 days. The temperature on the Moon varies with time, location, and crater depth. Around the equator, the temperature can reach 120 °C during the day and −130 °C when it is dark. The Northern and Southern Poles are much colder. For example, the North Pole has maximum temperatures ranging from −223 °C to 88 °C while minimum temperatures range from −253 °C to −173 °C [37]. Cables laid on top of the regolith would experience varying temperature swings along their lengths, making them vulnerable to accelerated thermal aging.

**Figure 4:**
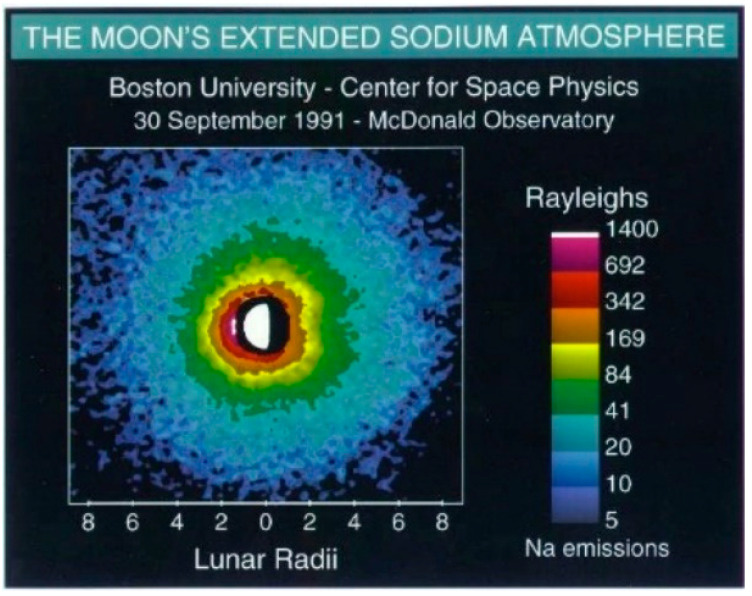
Glow from sodium in the lunar atmosphere.

It is often assumed that the Moon does not have an atmosphere. Although, it is near vacuum, what is not realized is that the Moon has a dusty plasma exosphere, evident by the “Lunar Horizon Glow” [46]. In December 1972, during the Apollo 17 Mission, the Lunar Atmospheric Composition Experiment (LACE) was deployed. LACE detected various atomic and molecular species [38,47]. The surface pressure is similar to that of low Earth orbit (LEO) at ~3 × 10^−15^ bar. The species detected at night included helium 4, neon 20 and 22, argon 40 and 36, carbon dioxide, and magnesium with traces of oxygen, aluminum, and silicon. Some of these gas species entrapped in the regolith could experience breakdown in the vicinity of conductors, as shown in Figure 1. During the day cycles, the atomic species rise due to thermal radiation from the sun so that the composition of the atmosphere is difficult to measure during the day. Figure 4 [47] shows Rayleigh light scattering of sodium in the lunar exosphere.

It is also clear that arcing even in LEO occurs at the same pressures as we might see on the Moon [7,48]. This is due to Earth’s free charged particles of the Ionosphere that are energized by the sun. There is also an electrical potential on the Moon, as discussed above. Based on Paschen’s curves, the breakdown of the regolith and the gaseous lunar exosphere, it can be assumed that electrical arcing and PD events can occur on lunar electrical assets, including high voltage electrical wiring, and cables. Until it is well understood, it is prudent to carry out research on energized lunar regolith or simulant, and insulation, to guide materials development.

### 2.3. High Voltage Operation in Space

**Figure 5:**
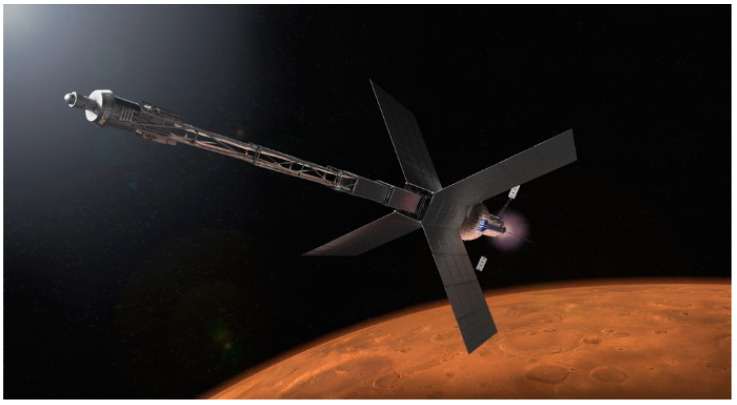
Illustration concept of a Mars nuclear propulsion system with transit habitat.

A mission to Mars is exciting and using nuclear fission power for exploration activities on the Moon, Mars and HV Nuclear Electric Propulsion (NEP) could one day be a reality [49,50,51]. The very nature of the journey leaves no room for error. Figure 5 [52] illustrates a NASA NEP spacecraft concept. The trip to Mars could sustainably reduce travel time if using advanced nuclear propulsion system either alone or combined with chemical propulsion [53]. These spacecraft systems are for manned long duration missions to Mars. The MW nuclear power likely requires HV operations (~1 KV) [51] and could significantly provide high performance without significantly increasing mass, compared to nuclear thermal propulsion [53].

Space environments outside of LEO can result in arcing events, as discussed previously. There are many challenges that fall under two environments for spacecraft. There is the external space environment, at near vacuum, and the internal spacecraft environment, due to variations of internal spacecraft pressures [7,13,51]. Operating in the space environment creates challenges for spacecraft related to the presence of micrometeoroids, vacuum, radiation and charged particles. These environments can cause damage to the spacecraft [7,13]. For example, high speed micrometeoroids can cause damage to exterior surfaces. The velocity of micrometeoroids can also cause debris to collect around energized connectors, causing premature electrical breakdown. Radiation can cause surface heating and damage electrical systems. Charged particles can sputter surfaces causing damage to thermal coatings and sensors. Additionally, charged particles can cause arcing events. High energy particles from solar flares and galactic cosmic radiation can interfere with spacecraft functions [7,13,54]. Thermal management of HV systems is a critical design parameter, since in a vacuum only radiant heat transfer is available. Inside the spacecraft heat can be transferred through conduction and can increase heat in electrical components, especially operating at HV and high frequencies. These challenges have been addressed by Dunbar and NASA [13,51], although new design guidelines may be necessary for voltages over 300 V, approaching kilovolt ranges.

Material from internal components in spacecraft, such as thermal insulation, face another high voltage challenge from electronic systems outgassing inside spacecraft due to external near vacuum operation. Although degassing is carried out on ground it is not necessarily 100% [54]. Depending on where the electrical systems are located, outgassing can cause arcing events, as described by the Paschen curve in Figure 1. Some materials entrap air or gases during processing or manufacturing of components. Outgassing can lead to gases escaping pin holes in compartments or gaps at cable interconnects. Materials are chosen with low outgassing properties. External spacecraft surfaces can outgas more rapidly than components that are surrounded by other materials or structures, such as thermal protection materials. This is due to the pressure differences throughout the external and internal spacecraft structure and components before and after launching. Once a spacecraft is launched, it takes a few minutes for the external pressure of the spacecraft to change from 1 bar to less 1 × 10^−5^ bar. The compartmentalized internal pressures can take a few minutes to 30 days to achieve the same exterior pressure, depending on degassing of surfaces or trapped air [7,13]. If HV operations begin at the critical minimum of pressure and electrode gap distance, arcing events occur that can significantly alter operations or cause mission failure. Voltages in the kilovolt range likely require robust lightweight materials and new design guidelines to minimize spacecraft weight and maximize HV performance.

## 3. Aerospace Electrical Insulation Research

For the past few years, researchers at NASA Glenn Research Center have been working on electrical insulation for HV megawatt class electrical insulation supporting two aeronautics mission programs: Transformative Aeronautics Concepts Program (TACP [7]) under the Transformational Tools and Technologies (TTT) Project and Advanced Air Vehicles Program (AAVP) under the Advanced Air Transport Technology (AATT) Project. In an effort to better understand research direction and goals, it is important to understand the current SOA aerospace insulation used in high altitude flight. SOA aerospace materials used for insulation are typically polyimides (PI) and fluoropolymers such as polytetrafluoroethylene (PTFE), perfluoroalkoxy alkanes (PFA), and fluorinated ethylene-propylene (FEP) [55]. Teflon-Kapton-Teflon (TKT) is a brand name for PTFE-PI-PTFE layered electrical insulation [56]. The layered TKT was designed to prevent moisture uptake in Kapton that leads to premature failure. Although these materials work well, due to their high dielectric strength, low arc tracking, flame retardancy, and ability to perform at high temperature [55,57], they are not necessarily suitable for HV application if tape wrapping is used to apply the 3 layered insulation onto the conductors. Extrudable electrical insulation is preferred to reduce the probability of internal defects that can lead to PD, thereby leading to degradation and failure [58]. Fluoropolymers are some of the best candidates for HV electrical insulation, due to good thermal stability, excellent electrical properties, and ease of extrusion [59].

These next sections, highlight the following NASA developed novel concepts: (1) micro-layered insulation structure, using various commercial PI layers bonded with PFA, (2) high thermal glass transition temperature polymer Polyphenylsulfone (PPSU) composites filled with commercial hexagonal boron nitride (h-BN) micro and nano fillers and (3) NASA-developed hexagonal boron nitride nanosheets (BNNSs). Voltage breakdown tests were carried out on the MMEI samples and the Polyphenylsulfone (PPSU) composites, according to ASTM D149-20, with a voltage ramp rate of 1.1 kV/s (AC) and 1.5 kV/s (DC) which are the minimum values on an Eaton Dielectric Test rig, Model DT2-60-20-SR-P-C, manufactured by Sefelec Eaton in France. The tests were carried out at 60 Hz and room temperature. MMEI samples were tested in air and in an insulation silicone oil. PPSU samples were carried out in air. Both MMEI and PPSU experimental work has been published before. For more detailed discussions on the MMEI, the PPSU composites and NASA-developed BNNS research, readers are directed to several recent publications [22,60,61,62,63,64,65,66,67,68]. This section captures highlights and provides an overview of some of the internal work that NASA GRC has been carrying out.

### 3.1. Micro-Multilayer Multifunctional Electrical Insulation (MMEI) System

The novel MMEI concept was conceived as part of the NASA-targeted CAS research strategy to understand how current commercially available SOA materials could be used today and what kind of HV performance could be achieved while targeting a lightweight system [22]. The MMEI concept was for future electric aircraft applications which critically require low weight but high voltage, high temperature, and corona or PD resistant insulation systems. Since then, its concept and feasibility were successfully demonstrated with its exceptionally high dielectric breakdown voltage by optimizing the multilayer structures of the Kapton^®^ PI layers and the binder layers, such as PFA, in terms of material type, individual layer thickness, and layer sequence [61,62]. Overall, MMEI structures outperformed most of the SOA polymer insulation materials or structures regardless of test environment or condition. A 19-layered MMEI structure [61] demonstrated a significant increase in voltage breakdown ~59 kV with a thickness of 0.54 mm, suggesting a ~98% reduction in insulation thickness when compared to Kapton^®^ PI at the same breakdown voltage. MMEI thicknesses ≤ 0.15 mm did not show any significant improvements over other SOA materials alone with similar thicknesses. Although the initial data indicated that the MMEI structures were very effective at higher voltages above 20 kV, their development is relevant for low voltage applications. This is because MMEI insulation de-rating factors have not yet been considered. De-rating factors include processing defects, electric stress, frequency, and temperature to name a few. This is important because the combination of HV and high frequencies (HF) are greater than those typically used. Materials operating at HV, and HF need to be investigated. Electric Aircraft could see frequencies between 1.5 kHz to 200 kHz [69,70,71]. Wire and cable de-rating factors reduce high breakdown voltages to significantly lower operation voltages [13]. In either case, the efforts were continued to maximize dielectric performance of the MMEI structures via material-design-process optimizations at minimum thickness. In addition, potential mechanisms responsible for MMEI performance were suggested and some have been validated experimentally via 3-dimensional dielectric breakdown damage and failure mode analyses. The MMEI structures induced a more torturous path for high voltage current flow through the insulation. Damage formation or propagation was effectively suppressed when decreasing the individual layer thickness, i.e., less chance of forming defects/voids in thinner film or smaller defects/voids, thus, less PD. It is worth noting that the MMEI structures could eventually take all the advantages of the Kapton^®^ materials, such as lightest weight, excellent physical–thermal–electric properties, excellent cut-through resistance and cold flow resistance, excellent radiation resistance, and low outgassing while mitigating Kapton’s disadvantages. Some of Kapton’s disadvantages include inflexibility, difficulty to strip, moisture absorption, degradation by atomic oxygen, poor weatherability, susceptibility to wet-arc and dry-arc tracking from abrasions and cuts, and instability to ultraviolet radiation.

**Figure 6:**
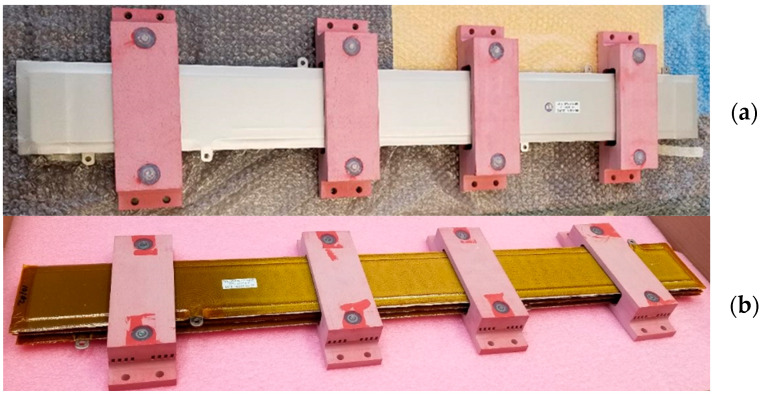
Full-scale 1 m long, 3-phase bus bar prototypes manufactured with the (**a**) with manufacturer’s SOA insulation and (**b**) MMEI system targeting 10 MW at 20 kV. A reduction in the average insulation weight (−15%) and thickness (−12%) was seen for the NASA MMEI prototype compared to the manufactured bus bar.

Significant progress in demonstrating scaling-up, manufacturability, and commercial applicability of the MMEI system has been made via full-scale prototype development, Figure 6. The bus bar was tested according to ASTM D19-20. PDIV was detected at 12 kV and breakdown occurred at 15 kV under 60 Hz AC as a quality control measure for actual applications. As seen in the figure caption, MMEI insulation was overall thinner and lighter than the insulation used by the manufacturer. MMEI performance will be further evaluated more extensively and systematically.

Multifunctionality was another major thrust for the MMEI development, for its structural concept and design ability. Important multifunctionalities needed for high voltage insulation systems in the electrified aircraft applications include PD resistance, electro-magnetic interference (EMI) shielding, moisture barrier, mechanical durability, thermal management and/or heat dissipation and so on. However, achieving the multifunctionality requires extensive and systematic material–design–process optimizations, as it is not simply about adding functional layers. Recently, a major effort has been initiated to modify and optimize the MMEI structures to significantly enhance PD resistance for high voltage power cable applications via incorporating semiconductive shielding layers and PD-induced degradation resistant nano materials.

Overall, the MMEI system demonstrated the use of micro-layers to achieve higher breakdown voltages, while reducing insulation thickness through careful design and processing methods insulation [11,72].

### 3.2. Next Generation in Aerospace Electrical Insulation Development

Second generation polymer insulators have evolved into materials that can provide enhanced thermal and dielectric characteristics, as well as resistance to aging. To address the needs of surviving under considerable thermal, mechanical, and electrical stresses, inorganic fillers were initially introduced to insulators to help provide performance enhancement in ways that pure polymeric insulators could not accomplish on their own. A comprehensive review of common types of inorganic fillers considered for high voltage insulators can be found in [73], which include SiO_2_, TiO_2_, Al_2_O_3_, ZnO, MgO, BN, AlN, Si_4_N_3_, and SiC. However, reasons differ as to why the dielectric properties of the composite insulator either improve or degrade once the filler is added to the host polymer [73]. Filler parameters, such as size, geometry, composition, relative permittivity, and conductivity are all believed to play a role in the resulting composite insulator’s dielectric properties, so the effects that these parameters play on insulation performance should be investigated and better understood in advanced dielectric materials.

Due to the extreme thermal environments anticipated in aerospace, high voltage applications, and high-performance polymers possessing superior thermal stability, as well as low relative permittivity are needed. As mentioned above, polyimides and fluoropolymers are usually the dominating polymer classes for such insulators; however, thermal management is a primary driver for next-generation aerospace insulators; therefore, heat dissipation characteristics are a property in dielectrics that cannot be overlooked.

The thermal conductivities of typical unfilled polyimides and perfluoroalkoxy resins are often measured to be below 0.25 W/m·K [74,75], which would imply that excessive filler volumes would have to be introduced to substantially increase thermal conductivity over that of the virgin polymer. Excessive filler volumes would ultimately lead to lower flexibility, higher density, and possibly lower dielectric properties, which is why new polymers should be considered.

Polyphenylsulfone (PPSU) is a tough, high-performance thermoplastic that is often used as a membrane material, as well as in interior aircraft cabin components. It has a starting thermal conductivity of ~0.33 W/mK and a glass-transition temperature of ~230 °C according to dynamic mechanical analysis. Alone, PPSU has poor UV stability. On the other hand, it is chemically resistant and inherently flame retardant and could, therefore, make a good insulation candidate if used with an additive to enhance UV durability. Boron nitride (BN) is a ceramic electrical insulator known for its radiation resistance, lubricious properties, high thermal stability and superior in-plane and through-plane thermal conductivities. For these reasons, incorporating h-BN with PPSU was investigated to assess its suitability as a composite insulation candidate in electrified aircraft [64].

**Figure 7:**
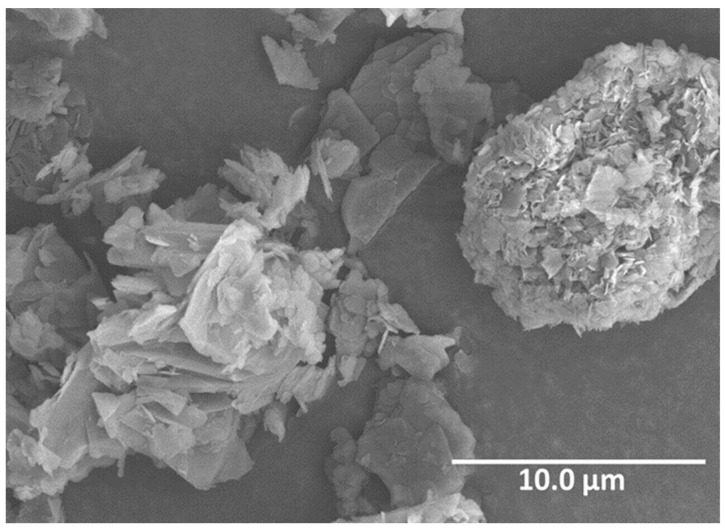
Scanning electron micrograph of commercially available, as received boron nitride micronized platelets. Magnification, 4.50 kX, working distance (WD): 12.5 mm.

Early trials extruding films of PPSU with commercially available h-BN, as shown in Figure 7, demonstrated a higher dielectric breakdown strength (DBS) in composites compared to the virgin PPSU and enhanced thermal conductivities [63,64]. More recent developments have revealed that gradual improvements in dielectric strength in both virgin PPSU and PPSU/h-BN composite films, have been made, compared to earlier experiments [63,64]. Data of recent studies given in Table 1 calculated according to [76].

**Figure 8:**
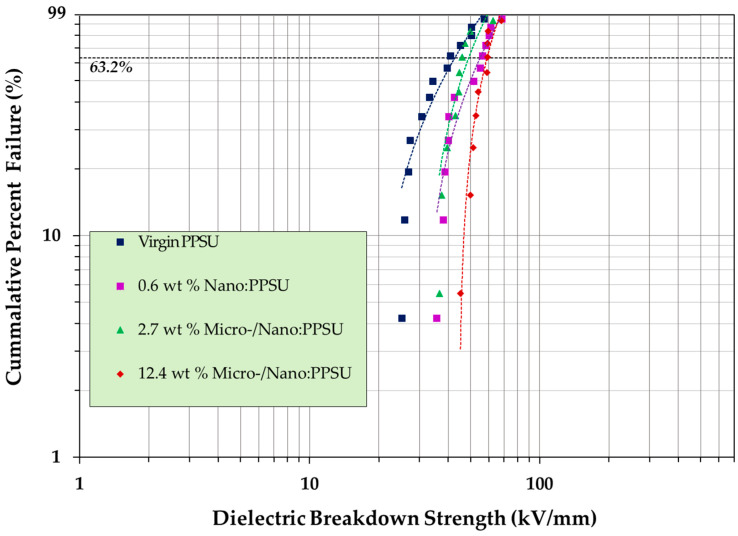
Weibull distribution plot of PPSU and PPSU/h-BN composites.

This behavior is illustrated in Figure 8, where the effect of the h-BN concentration on the dielectric strength of extruded virgin PPSU and PPSU-hBN composite films is shown in the Weibull plot. The data set was plotted according to IEEE/IEC 62539-2007 [76]. The extruded films in the batch had thicknesses between ~0.10–0.20 mm. At 63.2% failure probability, the 12.4 wt% PPSU-hBN composite had the highest breakdown strength out of all films at ~58.5 kV/mm. The shape parameter increased with higher BN filler concentrations, illustrating that the dielectric strength in composite samples were more consistent and had a narrower range of distribution. It was also observed that an exceptionally low filler of the nano h-BN composite resulted in the nanocomposite demonstrating the second highest dielectric strength among films in which h-BN was incorporated, illustrating the sensitivities of particle size, and controlling the matrix–filler interactions.

Preliminary data for PPSU and h-BN composites shows potential for use as an HV electrical insulator, but a significant amount of work should be carried out related to optimizing the extrusion process to obtain consistent film thickness, understanding the effects of h-BN and the composite microstructure on the partial discharge inception voltage, as well as the space charging. Additional work is underway, which includes understanding the aging mechanism of PPSU and h-BN composites. Aging and degradation behavior of high temperature polymers must be further investigated and could lead to the identification and development of effective stabilizers that can retard the aging response.

### 3.3. Hexagonal Boron Nitride for High Voltage Insulation Materials

Hexagonal Boron Nitride (h-BN) is one of the most significant ceramics today, due to its great properties and possibilities of application in many different industries, including Aerospace. The h-BN property list includes high mechanical rigidity, high thermal and chemical stability, high thermal conductivity, low dielectric constant and lightweight, among others [77]. The combination of these properties makes h-BN an exceptional multifunctional material for high voltage electrical system. However, working with h-BN has its challenges. Incorporating h-BN into a polymer matrix can be difficult and the improvement in properties in comparison with the bare polymer show that the multifunctional properties are diminished more than expected. One way to improve the properties of the h-BN/polymer composite is engineering h-BN to the application needs. For example, having thinner h-BN layers with high surface area while keeping high aspect ratio increases the contact area with the polymer and improves thermal conductivity, rather than just using thicker h-BN platelets. Furthermore, decreasing the layer numbers in h-BN can improve its thermal conductivity due to a reduced phonon–phonon scattering [77].

**Figure 9:**
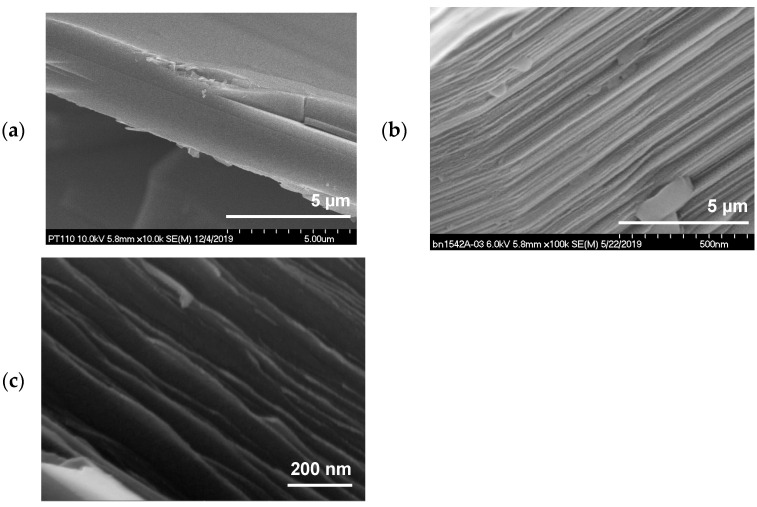
SEM micrographs of (**a**) commercial h-BN PT110, (**b**) h-BN PT110 intercalated with iron chloride, and (**c**) exfoliated h-BN (PT110).

NASA Glenn Research Center (GRC) developed a hexagonal boron nitride exfoliation process via intercalation of ferric chloride (FeCl_3_) to decrease the thickness and increase the surface area of commercial h-BN platelets [67,68,78]. This intercalation method was developed from the similarity between h-BN and graphite [79,80], the difference is that, due to boron nitride’s chemical stability, there is a need for an activation agent to force h-BN to react and allow the insertion of FeCl_3_ into the layers of BN [67]. The exfoliation of commercial h-BN with FeCl_3_ effectively decreases the thickness of the platelets, as shown in Figure 9. The scanning electron microscopy (SEM) micrographs show a commercial and unmodified h-BN PT110 manufactured by Momentive. Figure 9a shows the commercial h-BN PT110. Figure 9b shows the h-BN intercalated with FeCl_3_ that, later, was converted to iron oxide (Fe_2_O_3_) by a heat treatment in air. Figure 9c shows the exfoliated h-BN PT110 h-BN after Fe_2_O_3_ removal. The pictures show the sequential expansion process that takes place to the pristine h-BN with micron size thickness all the way to the exfoliated sample with thickness in the nanometer scale.

The exfoliation process can be modified and/or repeated as many times as necessary to get the desire properties. The more exfoliation cycles done, the thinner the h-BN until boron nitride nanosheets (BNNS) are obtained. Modification of the original intercalation/exfoliation process with FeCl_3_ has been conducted to engineer the product for other applications. One of the modifications is intercalating h-BN with aluminum chloride (AlCl_3_) [65], where, also, a coating of aluminum oxide (Al_2_O_3_) can be added to the surface of h-BN [65]. After the AlCl_3_ insertion into h-BN layers, it gets converted to Al_2_O_3_ during the exfoliation heat treatment process in air, and, contrary to the exfoliated samples with FeCl_3_, that are converted to Fe_2_O_3_ that can be dissolved in hydrochloridric acid (HCl), the Al_2_O_3_ cannot be dissolved and stays in/on the h-BN. Keeping Al_2_O_3_ intercalated and also coating the h-BN acts as a functional group and helps h-BN to mix better in a polymer matrix. Al_2_O_3_ has a thermal conductivity similar to through-plane h-BN, and it also has high heat resistance and chemical stability, making it compatible to use with boron nitride. These nano particles are being engineered to target desired electrical insulation properties for use as polymer fillers or other ceramic matrix composites. It is also important to note that the scalability of the engineered BNNS in the reactor has been demonstrated. Initial investigations yielded ~5 g of BNNS material per batch. NASA researchers have successfully scaled the yield to ~35 g per batch. It is anticipated that a scale up to 100’s grams per batch is easily achievable with a carefully engineered reactor.

## 4. Summary

Development of lightweight robust HV electrical insulation enables future high altitude MW class electrified aviation and supports NASA’s Lunar and Mars exploration Missions, while providing new Net Zero Emissions terrestrial transportation solutions. The research in the development of aerospace electrified propulsion assets has been going on for many years. However, there are gaps in research in the aera of HV aerospace electrical insulation materials for power transmission cables and wiring and other HV assets still under development. The current SOA cables and wires operating at higher voltages significantly increase the weight of an aircraft, payloads, and spacecraft.

The recent MMEI developments have demonstrated an alternative solution to traditional HV wiring and cables, in which an increase in insulation thickness would be required to accommodate the higher operating voltages. In this case, the overall cable weight can be reduced, resulting in significant weight saving when considering the hundreds and hundreds of miles of wires and cables used in aerospace systems. The PPSU h-BN composites and the patented MMEI and engineered h-BNNS provide viable solutions towards achieving a lighter weight electrical insulation.

Using new polymer composites, such as the PPSU and NASA engineered h-BN fillers, in the next generation of MMEI structured insulation could be revolutionary for the aerospace industry. The benefits are not limited to aerospace systems alone. The impact of these developments also provides lightweight solutions to HV terrestrial assets and, in particular, the ground transportation sector.

The authors hope that this paper inspires research activities across the world.

## Figures and Tables

**Table 1:** This table shows the sample names with h-BN weight percent, the sample thickness in millimeters, the mean voltage breakdown strength (BDS), the Weibull scale parameter α, the Weibull shape parameter β. The ^ indicates the weighted estimations of α and β.

Sample Names	Number of Samples	Thickness (mm)	Mean BDS (kV/mm)	$\hat{\alpha }$ (kV/mm)	$\hat{\beta }$
Virgin PPSU	13	0.17 ± 0.034	36.17	41.31	3.54
0.6 wt% NanoBN	13	0.20 ± 0.023	48.67	54.03	4.6
2.7 wt% micro/nanoBN	10	0.14 ± 0.019	44.68	47.68	6.79
12.4 wt% micro/nanoBN	10	0.10 ± 0.026	55.48	58.5	9.92

## Data Availability

The datasets used and/or analyzed during this study are available from the corresponding author on reasonable request.

## References

[1] Anderson C.D., Anderson J. (2010). Electric and Hybrid Cars: A history.

[2] Dorrington G.E. (2007). Performance of battery-powered airships. Proc. Inst. Mech. Eng. Part G J. Aerosp. Eng..

[3] Sahoo S., Zhao X., Kyprianidis K. (2020). A Review of Concepts, Benefits, and Challenges for Future Electrical Propulsion-Based Aircraft. Aerospace.

[4] (2019). NASA Aeronautics Strategic Implementation Plan 2019 Update.

[5] Madonna V., Giangrande P., Galea M. (2018). Electrical Power Generation in Aircraft: Review, Challenges, and Opportunities. IEEE Trans. Transp. Electrif..

[6] Nya B.H., Brombach J., Schulz D. Benefits of higher voltage levels in aircraft electrical power systems. Proceedings of the 2012 Electrical Systems for Aircraft, Railway and Ship Propulsion.

[7] Dunbar W.G. (1983). High Voltage Design Guide. Spacecraft.

[8] Borghei M., Ghassemi M. (2021). Insulation Materials and Systems for More- and All-Electric Aircraft: A Review Identifying Challenges and Future Research Needs. IEEE Trans. Transp. Electrif..

[9] Cotton I., Gardner R., Schweickart D., Grosean D., Severns C. Design considerations for higher electrical power system voltages in aerospace vehicles. Proceedings of the IEEE International Power Modulator and High Voltage Conference.

[10] Fang L., Cotton I., Wang Z.J., Freer R. Insulation performance evaluation of high temperature wire candidates for aerospace electrical machine winding application. Proceedings of the 2013 Electrical Insulation Conference.

[11] Dunbar W.G. (1983). High Voltage Design Guide. Aircraft.

[12] Khachen W., Suthar J., Stokes A., Dollinger R., Dunbar W.G. (1993). Aerospace-specific design guidelines for electrical insulation. IEEE Trans. Electr. Insul..

[13] NASA (2016). Spacecraft High-Voltage Paschen and Coroana Design Handbook.

[14] Cotton I., Nelms A., Husband M. (2008). Higher voltage aircraft power systems. IEEE Aerosp. Electron. Syst. Mag..

[15] Montanari G.C., Seri P., Naderiallaf H. (2020). A Contribution to Everlasting Electrical Insulation for DC Voltage: PD-Phobic Materials. IEEE Access.

[16] Montanari G.C. Bringing an insulation to failure: The role of space charge. Proceedings of the 2010 Annual Report Conference on Electrical Insulation and Dielectic Phenomena.

[17] Naderiallaf H., Seri P., Montanari G.C. (2020). Investigating Conditions for an Unexpected Additional Source of Partial Discharges in DC Cables: Load Power Variations. Trans. Power Deliv..

[18] Montanari G.C., Seri P., Bononi S.F., Albertini M. (2021). Partial Discharge Behavior and Accelerated Aging Upon Repetitive DC Cable Energization and Voltage Supply Polarity Inversion. IEEE Trans. Power Deliv..

[19] Dissado L., Mazzanti G., Montanari G.C. (1995). The incorporation of space charge degradation in the life model for electrical insulating materials. IEEE Trans. Dielectr. Electr. Insul..

[20] NASA (2013). N3-X blended wing body concept. NASA Turboelectric Concept Called the “N3-X,” with Blended Wing Body.

[21] Sadey D., Taylor L., Beach R. Proposal and development of a high voltage variable frequency alternating current power system for hybrid electric aircraft. Proceedings of the 14th International Energy Conversion Engineering Conference.

[22] Eugene Shin E.-S., Scheiman D.A., Lizcano M. Lightweight, durable, and multifunctional electrical insulation material systems for high voltage applications. Proceedings of the 2018 AIAA/IEEE Electric Aircraft Technologies Symposium (EATS).

[23] Lizcano M. Multilayered functional insulation system (MFIS) for AC power transmission in high voltage hybrid electrical propulsion. Proceedings of the EnergyTech.

[24] Rui R., Cotton I. Impact of low pressure aerospace environment on machine winding insulation. Proceedings of the IEEE International Symposium on Electrical Insulation.

[25] Sili E., Koliatene F., Cambronne J.P. Pressure and temperature effects on the Paschen curve. Proceedings of the 2011 Annual Report Conference on Electrical Insulation and Dielectric Phenomena.

[26] (2005). NASA/TM—2005-213629., T.W.K. Electric Power System Technology Options for Lunar Surface Missions.

[27] Khan Z., Vranis A., Zavoico A., Freid S., Nevada B., Manners B. (2006). Power System Concepts for the Lunar Outpost: A Review of the Power Generation, Energy Storage, Power Management and Distribution (PMAD) System Requirements and Potential Technologies for Development of the Lunar Outpost. AIP Conf. Proc..

[28] Thomas G., Granger M., Csank J., Gardner B. Establishing a lunar surface power grid. Proceedings of the 2022 Conference on Advanced Power Systems for Deep Space Exploration (APS4DS).

[29] NASA Manned Lunar Observatory Concept. https://www.nasa.gov/centers/glenn/multimedia/artgallery/lunar_observatory.html.

[30] (2017). Standard Specification for Concentric-Lay-Stranded Copper Conductors, Hard, Medium-Hard, or Soft.

[31] Rickman S., Johnson K., Maghsoudi E., Slenski G., Furst B., Wentzel D., Bautista A., Nelson E. (2018). Re-Architecting the NASA Wire Derating Approach for Space Flight Applications.

[32] DeMinico M., Csank J., Thomas G. (2022). Micro-grid Definition and Interface Converter for Planetary Surfaces (MIPS) Technical Assessment Periodic Review (TAPR).

[33] Jordan A.P., Stubbs T.J., Wilson J.K., Schwadron N.A., Spence H.E. (2015). Dielectric breakdown weathering of the Moon’s polar regolith. J. Geophys. Res. Planets.

[34] Yousif E., Haddad R. (2013). Photodegradation and photostabilization of polymers, especially polystyrene. SpringerPlus.

[35] Stubbs T.J., Vondrak R.R., Farrell W.M. (2007). Impact of dust on lunar exploration. Solar System.

[36] Khan-Mayberry N. (2017). The Lunar Environment: Determining the Health Effects of Exposure to Moon Dusts. https://ntrs.nasa.gov/api/citations/20070006527/downloads/20070006527.pdf.

[37] (2013). LUNAR RECONNAISSANCE ORBITER: Temperature Variation on the Moon.

[38] Williams D.R. Moon Fact Sheet. https://nssdc.gsfc.nasa.gov/planetary/factsheet/moonfact.html.

[39] Colwell J.E., Batiste S., Horányi M., Robertson S., Sture S. (2007). Lunar surface: Dust dynamics and regolith mechanics. Rev. Geophys..

[40] Oudayer P., Monnin L., Matéo-Vélez J.-C., Hess S.L.G., Sarrailh P., Murat G., Roussel J.-F. (2019). Multiscale Modeling of Dust Charging in Simulated Lunar Environment Conditions. IEEE Trans. Plasma Sci..

[41] Jordan A.P., Stubbs T.J., Shusterman M.L., Izenberg N.R., Wilson J.K., Hayne P.O., Schwadron N.A., Spence H.E. (2019). How dielectric breakdown may contribute to the global weathering of regolith on the moon. Icarus.

[42] Halekas J.S., Delory G.T., Lin R.P., Stubbs T.J., Farrell W.M. (2008). Lunar Prospector observations of the electrostatic potential of the lunar surface and its response to incident currents. J. Geophys. Res. Space Phys..

[43] Jordan A.P., Stubbs T.J., Wilson J., Schwadron N.A., Spence H.E. (2017). The rate of dielectric breakdown weathering of lunar regolith in permanently shadowed regions. Icarus.

[44] Jordan J.L., Heymann D., Lakatos S. (1974). Inert gas patterns in the regolith at the Apollo 15 landing site *Geochim*. Cosmochim. Acta.

[45] Eberhardt P., Geiss J., Graf H., Grögler N., Krähenbühl U., Schwaller H., Schwarzmüller J., Stettler A. (1970). Trapped Solar Wind Noble Gases, Kr^81^/Kr Exposure Ages and K/Ar Ages in Apollo 11 Lunar Material. Science.

[46] Richard D.T., Glenar D.A., Stubbs T.J., Davis S.S., Colaprete A. (2011). Light scattering by complex particles in the Moon’s exosphere: Toward a taxonomy of models for the realistic simulation of the scattering behavior of lunar dust. Planet. Space Sci..

[47] NASA Is There an Atmosphere on the Moon?. https://www.nasa.gov/mission_pages/LADEE/news/lunar-atmosphere.html.

[48] Anderson P.C. (2012). Characteristics of spacecraft charging in low Earth orbit. J. Geophys. Res. Space Phys..

[49] NASA A Lunar Nuclear Reactor. https://sservi.nasa.gov/articles/a-lunar-nuclear-reactor/.

[50] Cassady R.J., Frisbee R.H., Gilland J.H., Houts M.G., LaPointe M.R., Maresse-Reading C.M., Oleson S.R., Polk J.E., Russell D., Sengupta A. (2008). Recent advances in nuclear powered electric propulsion for space exploration. Energy Convers. Manag..

[51] Dyson R., Rao D.V., Duchek M., Harnack C., Scheidegger R., Mason L., Juhasz A., Rodriguez L., Leibach R., Geng S. Nuclear electric propulsion brayton power conversion working fluid considerations. Proceedings of the Nuclear and Emerging Technologies for Space (NETS-2022).

[52] NASA Nuclear Propulsion Could Help Get Humans to Mars Faster. https://www.nasa.gov/directorates/spacetech/nuclear-propulsion-could-help-get-humans-to-mars-faster.

[53] National Academies of Sciences, Engineering and Medicine (2021). Space Nuclear Propulsion for Human Mars Exploration.

[54] FAA. 4.1.2 Space Environemnts. https://www.faa.gov/about/office_org/headquarters_offices/avs/offices/aam/cami/library/online_libraries/aerospace_medicine/tutorial/media/III.4.1.2_The_Space_Environment.pdf.

[55] (2019). Wiring Aerospace Vehicle.

[56] Bettinger C. (2017). What Is TKT Insulated Wire?. https://www.interconnect-wiring.com/blog/tkt-insulated-wire/#:~:text=This%20wire%20has%20a%20tape,aircraft%20since%20the%20early%201990s.

[57] DuPont (2022). DuPont Kapton: Summary of Properties.

[58] Hähner T., Rybsky P., Cotton I., Lowndes R., Albert L., Thomas C., Dinculescu S., Teyssedre G. A Round-Robin test study of partial discharge inception voltage in aeronautic cables. Proceedings of the 2020 International Symposium on Electrical Insulating Materials.

[59] Ebnesajjad S. (2013). 6—Introduction to fluoropolymers. Introduction to Fluoropolymers.

[60] Shin E. (2019). High Performance Multilayer Insulation Composite for High Voltage Applications. U.S. Patent.

[61] Shin E.-S.E. Development of high voltage micro-multilayer multifunctional electrical insulation (MMEI) system. Proceedings of the 2019 AIAA/IEEE Electric Aircraft Technologies Symposium.

[62] Shin E.-S.E. Progresses in developing micro-multilayer multifunctional electrical insulation (MMEI) system for high voltage applications. Proceedings of the American Association for Advances in Functional Materials (AAAFM)-UCLA International Conference.

[63] Williams T., Nguyen B., Fuchs W. Polyphenylsulfone-hBN composite insulation. Proceedings of the IEEE 3rd International Conference on Dielectrics (ICD).

[64] Williams T.S., Nguyen B., Woodworth A., Kelly M. Engineered interfaces in extruded polyphenylsulfone-boron nitride composite insulation. Proceedings of the IEEE 4th International Conference on Dielectrics (ICD).

[65] Hung C.-C., Hurst J., Santiago D., Lizcano M., Kelly M. (2017). Highly thermally conductive hexagonal boron nitride/alumina composite made from commercial hexagonal boron nitride. J. Am. Ceram. Soc..

[66] Hung C.-C., Hurst J., Santiago D., Lizcano M., Kelly M. (2017). Synthesis and Thermal Conductivity of Exfoliated Hexagonal Boron Nitride/Alumina Ceramic Composite.

[67] Hung C.-C., Hurst J., Santiago D., Rogers R.B. (2014). Exfoliation of Hexagonal Boron Nitride via Ferric Chloride Intercalation.

[68] Hung C., Hurst J.B., Lizcano M., Santiago D. (2018). Compositions and Methods Associated with Intercalating and Exfoliating a Sample. U.S. Patent.

[69] Ivey C., Alfares A., He J. Hybrid-electric aircraft propulsion drive based on SiC triple active bridge converter. Proceedings of the 2021 IEEE/IAS Industrial and Commercial Power System Asia (I&CPS Asia).

[70] Dannier A., Brando G., Spina I., Raciti A., Rizzo S.A., Susinni G. High frequency converter topologies suitable for more electric aircraft. Proceedings of the 2018 AEIT International Annual Conference.

[71] Quinn P., Willis S. (2019). Measuring Techniques and Challenges of Moving to Variable Frequency Power.

[72] Tseng J.-K., Tang S., Zhou Z., Mackey M., Carr J.M., Mu R., Flandin L., Schuele D.E., Baer E., Zhu L. (2014). Interfacial polarization and layer thickness effect on electrical insulation in multilayered polysulfone/poly(vinylidene fluoride) films. Polymer.

[73] Saleem M.Z., Akbar M. (2022). Review of the Performance of High-Voltage Composite Insulators. Polymers.

[74] Li T.-L., Hsu S.L.-C. (2010). Enhanced Thermal Conductivity of Polyimide Films via a Hybrid of Micro- and Nano-Sized Boron Nitride. J. Phys. Chem. B.

[75] Zhang W., Zuo H., Zhang X., Wang J., Guo L., Peng X. (2018). Preparation of Graphene-Perfluoroalkoxy Composite and Thermal and Mechanical Properties. Polymers.

[76] (2007). Guide for the Statistical Analysis of Electrical Insulation Breakdown Data.

[77] Roy S., Zhang X., Puthirath A.B., Meiyazhagan A., Bhattacharyya S., Rahman M.M., Babu G., Susarla S., Saju S.K., Tran M.K. (2021). Structure, Properties and Applications of Two-Dimensional Hexagonal Boron Nitride. Adv. Mater..

[78] Hung C.C., Hurst J. (2018). Methods for Intercalating and Exfoliating Hexagonal Boron Nitride. U.S. Patent.

[79] Hod O. (2012). Graphite and Hexagonal Boron-Nitride have the Same Interlayer Distance. Why?. J. Chem. Theory Comput..

[80] Gautam C., Chelliah S. (2021). Methods of hexagonal boron nitride exfoliation and its functionalization: Covalent and non-covalent approaches. RSC Adv..

